# Aggressive Pseudomyxoma Peritonei: A Case Report with an Unusual Clinical Presentation

**DOI:** 10.1155/2013/926963

**Published:** 2013-11-13

**Authors:** Zisis Touloumis, George Galyfos, Nikolaos Kavouras, Michalis Menis, Laurant Lavant

**Affiliations:** Department of General Surgery, General Hospital of Chalkis, 48 Gazepi Street, Chalkis, 34100 Evia, Greece

## Abstract

*Introduction*. Pseudomyxoma peritonei (PMP) is an uncommon surgical entity. We report a case of aggressive disease with an unusual clinical presentation and we analyze current data on diagnosis and management of PMP. *Case Presentation*. A 71-year-old male patient presented with intermittent diarrhea and loss of appetite during the last two months, without any other classic symptoms of PMP. The clinical examination was misleading due to patient's obesity. The radiological evaluation revealed ascites of the abdomen and possible mucocele of the appendix, whereas the laboratory exams showed high values of specific tumour markers. The patient underwent an exploratory laparotomy for definite diagnosis. Biopsies and immunohistochemical examination confirmed the diagnosis of an aggressive and extended peritoneal mucinous carcinomatosis (PMCA). The patient was programmed for adjuvant systematic chemotherapy, which was not completed due to progression of the disease. *Conclusions*. Progressed PMP can present with unspecific symptoms that mislead diagnosis. Cytoreductive surgery in combination with systematic chemotherapy could be appropriate for aggressive PMCA, even with an unfavourable prognosis.

## 1. Introduction

Pseudomyxoma peritonei (PMP) is an uncommon tumor known for its production of mucin in the abdominal cavity. If left untreated, mucin will eventually build up to the point where it compresses vital structures, such as the colon, the liver, kidneys, stomach, spleen, and pancreas [1]. For years, the clinical syndrome of PMP has been enigmatic. The term PMP was first introduced by Werth in 1884 and today, the overall incidence is estimated to be ~1-2 per million per year [1, 2]. 

Regarding its classification, PMP is a broad descriptive term embracing a wide spectrum of biological behaviour of neoplasms, from the benign to the frankly malignant lesion. Ronnett and colleagues proposed a classification distinguishing “disseminated peritoneal adenomucinosis” (DPAM) from “peritoneal mucinous carcinomatosis” (PMCA) [3]. Furthermore, DPAM represents the classic PMP with paucicellular mucinous ascites and an indolent clinical course, whereas PMCA has a higher percentage of overtly malignant cells/cell groups and a poorer prognosis [4]. There is increasing recognition that the two variants of PMP-DPAM and PMCA are different, with the DPAM type remaining localized to the abdomen without metastatic behaviour and the PMCA type behaving like a mucinous (colloid) carcinoma with metastatic and invasive potential [3, 4]. 

Regarding the clinical presentation, pseudomyxoma peritonei is a disease more commonly seen in women (male : female ratio = 9 : 11), with an average age of 53 years who usually present with increasing abdominal girth and a primary ovarian lesion [1, 5]. Though uncommon in men, male cases with PMP are all virtually associated with a lesion in the appendix [1, 5, 6]. Other possible primary sites include colorectum, gallbladder, pancreas, urachus, urinary bladder, breast, and lung, but these are uncommon. Moreover, PMP can occur years (ranging from 5 to 35 years) after the initial presentation of an appendiceal event and, therefore, diagnosis prior to surgery is often delayed and inaccurate [6, 7]. Additionally, 10% of patients die of PMP within 5.5 years of their initial presentation. Overall survival of patients is about 75% and 68% for 5 years and 10 years, respectively, as revealed by Ronnett et al. [3, 4].

The usual clinical features of this tumor are increasing abdominal girth (40%), bilateral or unilateral ovarian tumors (20%), hernia sac tumors (20%), appendicitis-like syndrome (10%), and infertility (10%) [1, 5, 6]. Narrowing, but rarely complete obstruction, of the gastrointestinal tract frequently occurs at three well-defined anatomic sites—the pyloric antrum, the ileocecal valve, and the cul-de-sac of Douglas [1, 2, 5]. These are three portions of the gastrointestinal tract that are attached to the retroperitoneum and are relatively motionless. Although intestinal obstruction is rarely reported as an initial manifestation of this disease, it usually occurs when multiple previous surgeries have led to small bowel entrapment [8]. The disease may be localized in the right lower quadrant initially and then become more generalized with mucinous peritoneal, serosal, and ommental implants [6–8]. 

Proper diagnostic investigations include an ultrasonographic examination of the abdomen initially, whereas computed tomography scans will reveal further information about the extent of the disease [9]. Additionally, the evaluation of tumor markers in serum, such as Ca 19-9 and carcinoembryonic antigen (CEA), shows a prognostic role [10]. This disease is most often discovered during surgery for other conditions, for example, hernia repair, following which an experienced pathologist can confirm the diagnosis. Due to the rarity of PMP, it is important to obtain an accurate diagnosis so that appropriate treatment may be obtained.

We report a case of PMCA in a male patient with an unusual clinical picture, who underwent an exploratory laparotomy and was programmed for systemic chemotherapy afterwards.

## 2. Case Presentation

Male patient, 71 years old, presented to the emergency department complaining of episodes of intermittent diarrhea without any fever, vomiting, or abdominal pain. The symptoms had appeared, for the first time, two months prior to the patient's presentation. During the clinical examination, there were no palpable masses of the abdomen and an abdominal distention could not be clearly diagnosed, due to patient's obesity. The patient complained also about loss of appetite during the last two months. 

Regarding the standard serum investigations, we observed anemia (HCT 30%), without any other abnormal values of biochemical tests. There was an ultrasound requested, if there was a significant amount of intraperitoneal ascites discovered. Tumor markers were also measured in serum, where Ca 19-9 and CEA serum levels were higher than normal values (Ca 19-9 = 204.4 IU/mL and CEA = 83.5 ng/mL). The computed tomography (CT) control of the abdomen showed that the peritoneal cavity included a significant amount of ascites, and a cystic mass (8 cm in diameter), with small calcifications within its wall, was found in the right iliac fossa, possibly a mucocele of the appendix. Moreover, no enlarged lymphnodes of the abdomen were observed. Colonoscopy and gastroscopy findings were negative for pathology. A small amount of ascitic collection was drawn percutaneously for cytologic examination. The result was a small number of observed lymphocytes in the setting of large amount of mucous matrix. There were no malignant cells observed. 

The patient underwent an exploratory laparotomy for definite diagnosis. The abdomen was full of gelatinous ascitic liquid and diffuse implantations of the peritoneum. Extended adhesions of the small intestine to the abdominal wall due to neoplastic implantation were found as well. A large mucocele was identified arising from the appendix. The disseminated picture of the disease indicated a cytoreductive surgery procedure. Biopsies were taken from the abdominal wall and the mesenterium. They revealed elements of peritoneal mucinous neoplasm of high malignant grade and microscopic lesions of low differentiated character. The histopathology and further immunoassays (CK7 negative and CK20 positive) confirmed the diagnosis of a disseminated PMCA of appendiceal origin. The patient underwent 4 cycles of systematic chemotherapy (5-fluorouracil based) as well, which was interrupted due to secondary toxic effects and progression of the disease in the abdomen.

## 3. Discussion

In the past, pseudomyxoma peritonei (PMP) has been attributed to a variety of primary tumors [1, 2]. This may be true, but, in the vast majority of cases, the patients have an appendiceal tumor giving rise to this clinical entity, as was the case with our patient. Recently, the increased usage of immunohistochemical stains and molecular genetic studies has shown that this happens in both men and women [1, 11]. In women, most cases of ovarian involvement are favored to be a metastasis from an appendiceal source or another gastrointestinal source [12]. This is the reason why in many institutions, it has become a standard procedure to perform an appendectomy routinely during the staging of ovarian neoplasms [13]. However, mucinous peritoneal carcinomatosis may arise from other sites, but these tumors usually have signet ring histology [1, 12]. They may show redistribution but do not spare the small bowel and will implant and grow in the abdominal cavity in a random fashion with extensive small bowel involvement, resulting in a much poorer prognosis [14]. Unlike most cancers, this disease rarely spreads through the lymphatic system or through the bloodstream. Therefore, it is characterized by mucin and scattered cancer cells in the abdominal cavity, as it was observed in our case.

As mentioned above, PMP has multiple clinical manifestations that lead to difficulties in definitive diagnosis and timely treatment [6]. As symptoms remain nonspecific, the disease presents a great diagnostic challenge to clinicians. Patients usually experience a long course of health deterioration before an accurate diagnosis is made [5, 6]. The main symptoms of our patient were misleading as well and delayed the final diagnosis. Additionally, none of previous reports have included diarrhea as a typical symptom of PMP. In our case, a cystic mass of great size was located in the region of the ileocecal valve causing probably incomplete obstruction of the right colon. Furthermore, during the laparotomy, extended adhesions of the small intestine to the abdominal wall were discovered. All the above data point out a possible pseudoobstructive syndrome, characterized by diarrhea as the main manifestation. Diagnosis of the disease was confirmed through pathology. A definitive diagnosis of PMP requires the presence of (a) mucinous neoplastic cells/epithelium and (b) mucinous ascites—diffuse intra-abdominal mucin [1, 2]. Some authors also require the presence of diffuse mucinous implants for this diagnosis [3, 6]. Viable epithelial glandular cells must be identified within the mucin pools by histological analysis to diagnose PMP. The biopsy of our patient concurred. Cases without epithelium are regarded as mucinous ascites. 

Regarding the preoperative evaluation of possible biomarkers, PMP patients with preoperative elevated tumor markers such as CEA and Ca 19-9 are at increased risk of developing recurrent disease despite aggressive therapy [15]. Likewise, PMP patients with normal levels of these tumor markers have an overall improved prognosis. In the recent study by Canbay et al., researchers concluded that preoperative CEA levels are useful in predicting the extent of disease and surgical success as well as progress-free and overall survival in patients with PMP treated with cytoreductive surgery and hyperthermic intraoperative peritoneal chemotherapy (HIPEC) [16]. These results agree with our case, where a progression of disease was observed postoperatively, given the high CEA and Ca 19-9 serum levels preoperatively. The prognosis of PMP is closely related to the bulk of the disease as evaluated by the tumor site, preoperative tumor volume and completeness of tumor removal by cytoreductive surgery, and the microscopic degree of differentiation of the neoplastic epithelium as evaluated by the histopathological examination [17]. The highly malignant character of the disease (high serum markers plus high tumor volume intra-abdominally) in our case leaded to early progression of the disease.

Treatment of PMP is variable, both due to the rarity of the disease and to its frequently slow-growing nature [1]. Current treatment strategies range from watchful waiting to cytoreductive surgery with HIPEC or early postoperative intraperitoneal chemotherapy (EPIC) [18, 19]. Based on the Sugarbaker peritonectomy procedure, a study by Deraco et al. showed that cytoreductive surgery with intraperitoneal hyperthermic perfusion permitted complete tumor removal, and this study confirmed the efficacy of this combined treatment in terms of improved long-term survival and better local control of the disease [20]. In situations where surgery is not required immediately, patients can be monitored via CT scans, tumor marker laboratory tests, and physical symptoms, to determine when, and if, surgery is warranted.

Likewise, regarding the proper management of aggressive forms of PMP, there is still a controversy among the researchers. Recent studies support that cytoreduction with peritonectomy plus HIPEC is a safe procedure that suggests an improvement to the survival rates, even in aggressive cases [21, 22]. However, the authors in the recent study by Faris and Ryan conclude that the treatment of the low grade variants of PMP includes serial cytoreduction surgery, with data indicating possible, but unproven, benefit from HIPEC, whereas there is no consensus so far on the role of cytoreduction and HIPEC for the management of the more aggressive histological variants and peritoneal carcinomatosis [23]. As a result, they support that systemic chemotherapy should be the standard of care for patients with the high grade variants and peritoneal carcinomatosis, as in our case. Recent studies show that a fluorouracil-based adjuvant chemotherapy can be used for PMP of appendiceal origin and the results are promising [24]. However, one must hence know that most of these studies do not focus on cases of aggressive PMP. 

Finally, pseudomyxoma peritonei may recur following cytoreductive surgery and systemic chemotherapy, as seen in our case, especially when the disease is diagnosed in an advanced stage [25]. Periodic post-operative CT scans and tumor marker evaluation should be used to monitor the disease for any tumor regrowth. Furthermore, clinical awareness and recognition of PMP as a potential delayed consequence years later after an appendicectomy should alert all surgeons to be extremely vigilant while treating mucinous neoplasms of the appendix, with special care being directed towards adequate excision and thorough debridement at the initial diagnosis. 

## 4. Conclusions

We have described an unusual case of aggressive and progressed PMCA, with a misleading clinical presentation. There is no consensus regarding the proper management of aggressive cases. Cytoreductive surgery in combination with systematic chemotherapy could be appropriate for aggressive PMCA, even with an unfavourable prognosis.
